# The Impact of Environmental Commitment on Green Purchase Behavior in China

**DOI:** 10.3390/ijerph19148644

**Published:** 2022-07-15

**Authors:** Xixiang Sun, Ziyuan Tian, Jianguo Wang, Weihuan Su

**Affiliations:** School of Management, Wuhan University of Technology, Wuhan 430070, China; 13971190718@163.com (X.S.); jgwang@whut.edu.cn (J.W.); suweihuan@whut.edu.cn (W.S.)

**Keywords:** environmental commitment, green purchase behavior, anticipated pride, anticipated guilt, social norms

## Abstract

There is no consensus on whether environmental commitment can promote green purchase behavior. Especially in the Chinese context, the validity and internal mechanism of the impact of environmental commitment on green purchase behavior have not been deeply studied. Based on Norm Activation Theory and Theory of Planned Behavior, this study explores the influence mechanism of environmental commitment on consumers’ green purchase behavior. Research data were derived from China, and analyzed using randomized control trials. Results reveal that environmental commitment significantly promotes green purchase behavior. Anticipated pride and anticipated guilt mediate the relationship between environmental commitment and green purchase behavior. Social norms moderate the positive effect of environmental commitment on anticipated pride and anticipated guilt. Compared with low social norms, environmental commitment has a greater effect on anticipated pride and anticipated guilt in the case of high social norms. This study provides new insight into environmental commitment and how to promote green purchase behavior, and the findings could help governments and marketers to formulate future policies and strategies to promote consumers’ green purchase behavior.

## 1. Introduction

Rapid economic development and technological advancement have brought more convenience to people’s lives, but also pose many threats to the environment, such as air pollution, climate change, and global warming. These problems directly affect the sustainability of economic development, the environment, and society [1]. Relevant research showed that 30–40% of the deterioration of the ecological environment was caused by personal and household consumption [2]. The government’s environmental protection measures and the positive response of enterprises can promote the development of a green consumption mode to a certain extent, but as the terminal of green consumption, the consumers’ life and consumption mode are the top priority in promoting green consumption. Therefore, transforming the consumption pattern into green consumption with less negative effects on the environment is significant for realizing the harmonious coexistence of humans and nature [3]. The public pays more attention to environmental protection and gradually tends to increase their consumption of green products. Especially during the COVID-19 pandemic, public perceptions and behavior patterns were altered in a safer, healthier, and greener direction [4,5]. Accordingly, the production and sale of green products have received attention from enterprises. Business managers and scholars have been seeking for ways to encourage consumers to buy green products [6].

Environmental commitment is defined as psychological attachment to and long-term orientation toward the natural world [7]. Some scholars have found that the improvement of consumers’ environmental commitment can effectively promote their green purchase behavior. For example, Hojnik et al. [8] have demonstrated that consumers’ environmental commitment has a positive impact on green purchase intention, thus leading to green consumerism (i.e., an actual purchase or environmentally friendly behavior). However, other scholars have obtained different conclusions. The research results of Shen et al. [9] have shown that the level of environmental commitment failed to predict the willingness to pay an entrance fee for environment conservation and protection. The following reasons may explain this inconsistency: (1) Different samples. Based on the national environment, Hojnik et al. [8] randomly selected 705 consumers living in Slovenia and speaking the local language as respondents. While based on a particular site, Shen et al. [9] randomly selected 370 visitors in Yangmingham National Park as respondents. The reason could be that Yangmingshan National Park has environmental goods, a subset of public goods, where the individual who pays more money for protection or conservation cannot prevent other environmental users from using the natural site without paying anything. Although committed, the willingness to pay for conservation would not be increased [10,11]. (2) The above two research studies only studied the direct impact of environmental commitment on green purchase behavior, both ignoring the internal influence mechanism. Based on different cultural backgrounds, the internal influence mechanism of environmental commitment on green purchasing behavior may be different, thus resulting in inconsistent results. Our study aims to explore the internal mechanism of the impact of environmental commitment on consumers’ green purchase behavior in China, which is an effort to partly fill the above gaps, confirming the effect of sample selection on the results to a certain extent. Moreover, in recent years, most research studies on green purchasing were concentrated in developed countries, with limited research conducted in developing countries [12]. At present, China’s green consumption market is still immature. Although consumers’ environmental awareness is constantly improving, consumers’ actual green purchase level is not high, which seriously lags behind the development of China’s green consumption market. Therefore, it is of great significance to study the internal influence mechanism of environmental commitment on consumers’ green purchase behavior in China.

The theory of planned behavior proposes a general framework within which the private behaviors of consumers can be understood. Therefore, the determinants of subjective attitudes and behaviors can be identified through this theory to predict consumers’ intentions so that their intentions measure the actual behavior of customers [13]. According to this theory, intentions are seen as the basis that evaluates the orientation and behavior of consumers and their level of readiness [14]. This theory explains that consumers’ attitudes towards their actual actions depend primarily on their personal behavioral belief in the product with the knowledge-based assessment of the decision to purchase this product. Thus, these attitudes may be influenced by personal criteria related to more objective specifications of belief in consumption and the consumer’s purchase motivations [15]. Many previous pro-environmental studies of green product purchases have used the theory of planned behavior (TPB) to examine the behavior associated with such purchases [16].

The normative activation perspective proposes transforming ethical standards into environmental behavior [17], as consumers’ willingness towards protecting the environment drives them to environmentally friendly and sustainable products [18], thus promoting green social norms through green purchase behavior [19]. This theory holds that anticipated guilt and pride cause people to behave in line with known personal norms. According to this theory, many fundamental variables determine consumers’ intentions towards environmentally friendly products, such as normative beliefs, perceived behavioral control, awareness of the consequences of environmental problems, and intention to act in an environmentally friendly way.

Based on Norm Activation Theory and Theory of Planned Behavior, this study uses randomized control trials to explore the internal mechanism of the impact of environmental commitment on consumers’ green purchase behavior in China. Meanwhile, it takes into account the internal emotional factors of consumers—anticipated pride and anticipated guilt—and an external factor—social norms—to make the content more in accordance with realistic situation. This study provides new insight into exploring the internal mechanism of the environmental commitment affecting consumers’ green purchase behavior. Additionally, the findings can help governments and green product enterprises to formulate relevant policies and marketing strategies to promote green purchase behavior.

## 2. Literature Review and Hypothesis Development

### 2.1. Green Purchase Behavior

Green purchase behavior refers to purchasing environmentally friendly products that can be recycled, which is advantageous to the natural environment [20]. A large number of studies have shown that the motivation for green purchase behavior is gradually increasing [21,22,23]. Previous studies have shown that the factors affecting green purchase behavior mainly include the following four aspects:

(1) Cognitive, Psychological, and Sociodemographic

Studies of the determinants of green purchasing regarding cognitive, psychological, and socio-demographic factors are the most commonly found in the literature. Chan and Lau [24] examined the impact of ecological knowledge, cultural values, and environmental affect; their results showed that there is a positive effect of the three factors, with great emphasis on environmental knowledge and the ecological impact on consumer’s intention to buy green products, so that the more the consumer enjoys environmental knowledge, the greater their orientation towards purchasing green products. The study by Lee [25] discussed the effect of gender on enhancing purchase intentions; the results of his research showed that females tend to buy green products more than males. It should be noted that the studies that discussed the personal characteristics of the consumer, such as age, gender, education, knowledge, and income level, came first, which confirms the association of green purchasing with the theory of planned behavior, which has been broadly used in the literature.

In addition, we have noticed that there has been a development in studies in that they have begun discussing the demographic factors directly related to the consumer. In some recent studies, they started using other characteristics, such as Liang et al. [26], who investigated pride, gratitude, guilt, and condemnation of others as the main factors in determining a consumer’s intention to buy green products. The study showed that the feelings of pride and gratitude, as factors that fall within the positive emotions of the consumer, enhance the purposes of green purchasing. On the other hand, negative feelings, such as the condemnation of others and guilt, moderate purchase intention. The study concluded that the availability of purposes related to avoiding environmental pollution enhances the sense of green purchasing.

On the other hand, some studies have differences in determining the influence of factors, whether positive, negative, or no effect, such as Jaini et al. [27], in which the result of their study showed that altruism has no impact, while the study by Jaini et al. [28] found that it impacts beliefs that support the environment, which ultimately affects the consumer’s personal behavior.

(2) Product Attributes

Some research findings suggest that price and packaging are among the most significant factors that encourage consumers to purchase green products. Weisstein et al. [29] investigated the effect of the product’s price on the tendency to buy green products. The results showed that different offers for product prices positively affects the intention and perceptions of consumers to purchase green products, as promotional offers that result in gains affect consumers interested in green products. In contrast, consumers who do not enjoy a high degree of green are attracted to promotional offers that decrease losses.

Martinho et al. [30] investigated the effect of the packaging of a product on the propensity to purchase green products. The results showed the presence of two groups of consumers: the first group is interested in packaging, whereas the second group considers packaging nonessential when making a purchase decision. Other studies discussed the role of other factors in marketing green products, such as advertising [31].

(3) Consequence Design of Products

To attract consumers to buy environmentally friendly green products, there must be a different design. Product design is one of the main factors contributing to promoting green products, such as sustainable designs, which express the philosophy of designing products and services compatible with the principles of environmental sustainability. Martinho et al. [30] believed that a large group of surveyed consumers showed an interest in sustainable, environmentally friendly packaging. Therefore, one of the essential points that may encourage the consumer to buy green products is the perceived intensity at the moment of purchase over packaging and design, which is more critical than the actual input intensity [32]. Maslow’s theory shows that consumers who are self-sufficient in basic needs always seek satisfaction in achieving sufficiency from other conditions, such as material and luxury needs associated with sustainability [33]. Thus, the importance of product design in a way that shows environmental protection may enhance the attraction of consumers to purchase. It can be said that there are different ways through which advertising calls to buy green products can be practiced, such as describing the features and characteristics of the product in a more subjective or general way, or describing the attributes and characteristics of the product in an objective and detailed manner through tangible attractiveness [34]. According to a study by Yang et al. [35], advertising related to tangible attractiveness is less effective than the abstract attractiveness method based on describing the product in general or subjectively when the characteristics and attributes of green products are related to consumers and their interests. Therefore, companies are obligated to clarify the importance of environmental protection for green products and show the company design to show their value and personality in a way that distinguishes between traditional products and green products. On the other hand, the results of some studies indicate that product design may significantly enhance competition, survival, and continuity in the market and determine the company’s success by identifying the need and satisfying consumers’ desires [36].

(4) Social and Environmental Factors

Dagher and Itani [37], who discussed the impact of perceived effectiveness of environmental behavior, sensed environmental responsibility, perceived seriousness of environmental issues, and concern for self-image in environmental protection on green purchasing choices. The study results showed an effect of perceived environmental responsibility, perceived seriousness of environmental problems, and concern for self-image in environmental protection green purchasing intentions. On the other hand, the results showed no effect of perceived effectiveness of environmental behavior on green purchasing intentions. The results of a study by Uddin and Khan [38] found a positive impact on the perceived usefulness of environmental behavior on green purchasing, as the evidence provided by Visser and Dlamini [39] showed no significant effect of environmental attitude and environmental knowledge on green purchase intentions. On the contrary, the study results by Goh and Balaji [40] showed that environmental knowledge plays a significant mediating role in promoting green purchasing intentions. What is more, some scholars believe that consumers’ environmental commitment has a positive impact on their green purchase behavior [8,41,42], while others suggest that environmental commitment cannot predict consumers’ green purchase intention [9].

Therefore, there is no consensus about whether environmental commitment can promote consumers’ green purchase behavior. Currently, the relevant studies mostly focus on whether environmental commitment has an impact on consumers’ green purchase behavior [8,9,41,42], with a clear lack of research on its internal influence mechanism. Additionally, in recent years, most research studies on green purchasing were concentrated in developed countries, with limited research conducted in developing countries [12]. Therefore, it is of great importance to study the internal influence mechanism of environmental commitment on consumers’ green purchase behavior in China.

### 2.2. Environmental Commitment and Green Purchase Behavior

Environmental commitment is psychological attachment to and long-term orientation toward the natural world [7]. Green purchase behavior is a pro-environmental behavior since the action enhances the quality of the environment through purchase decisions that reduce environmental problems arising from unsustainable production and consumption [43]. Previous research has demonstrated that environmental commitment promotes consumers’ pro-environmental behavior. Rahman and Reynolds [41] found that consumers’ environmental commitment significantly affects their choice of hotels, and consumers with high environmental commitment tend to choose green hotels. In Hergesell’s study [42], travelers with high environmental commitment tend to choose public transportation during their travels; they pay more attention to the impact of their behavior on the environment. Liu and Lin [44] believed that Taiwanese college students with higher environmental commitment show more attention and greater willingness to protecting the environment. Davis et al. [45] pointed out that individuals with high environmental satisfaction and investment are more likely to have high environmental commitment, which further urges them to engage in environmentally friendly behaviors. Terrier and Marfaing [46] suggested that environmental commitment tends to strengthen individuals’ perceptions of themselves, thus motivating them to become environmentally friendly individuals. Therefore, an individual’s environmental commitment is a significant factor influencing green purchase behavior that is beneficial to the environment. When individuals have higher environmental commitment, they can feel a closer relationship with nature, and realize that their behaviors have an impact on the environment, thereby choosing to follow a more environmentally friendly consumption behavior, such as green purchasing. Hence, we propose the following hypothesis:

**Hypothesis** **1** **(H1).** *Environmental commitment is positively associated with green purchase behavior*.

### 2.3. The Mediating Role of Anticipated Pride and Anticipated Guilt

When making decisions, people often anticipate how they will feel about future outcomes and use those feelings as guides to choices. Anticipated emotions are more intense than those actually experienced afterwards [45]. Studies have found that anticipated emotions affect behaviors [47], because individuals will strive to experience positive emotions and avoid negative ones [48].

Pride and guilt, known as self-conscious emotions [49,50], arise from evaluations of oneself after following (or failing to follow) personal or social standards [49]. These personal and social standards are often based on moral behavior, and the subsequent self-conscious emotions elicited by these standards stimulate altruistic behavior [51]. Current research maintains a discrete emotional perspective [52]; that is, pride and guilt are not two extremes of one emotion, but two different emotions that need to be measured separately.

Previous research has shown that emotions influence individuals’ pro-environmental behavior. For example, Mi et al. [53] studied the impact of the COVID-19 epidemic on the public’s pro-environmental behavior and found that positive emotions and negative emotions as mediating variables can significantly promote the public’s pro-environmental behavior. Mi et al. [54] argued that the anticipated pride and anticipated guilt can obviously affect employees’ pro-environmental behavior in the workplace. Bissing-Olson et al. [55] put forward that pride has a significant positive impact on pro-environmental behavior. Rosenthal and Ho [56] confirmed that anticipated negative emotions positively influence residents’ pro-environmental behaviors, such as littering or picking up garbage. Liang et al. [26] demonstrated that positive emotions (such as pride) and negative emotions (such as guilt) can have a significant effect on consumers’ green purchase behavior.

In Norm Activation Theory, personal norms represent an internalized behavior standard. Individuals will feel proud when they follow personal norms, and guilty when they violate personal norms [57]. Environmental commitment, as an embodiment of personal norms in the pro-environmental domain, stimulates individuals to form anticipated pride and anticipated guilt. For consumers, the higher the environmental commitment, the more they will be aware of the close connection between themselves and the natural environment, and thus be more aware of environmental protection and the impact of green purchase behavior on the environment. When consumers expect that they implement green purchase behavior, they realize that they will do something beneficial to the environment, thus resulting in a stronger sense of anticipated pride. In the case of non-green purchase behavior, consumers realize that their consumption behavior will have a certain adverse impact on the environment, thus resulting in a stronger sense of anticipated guilt. All of these will promote consumers to adjust their emotions, increase positive emotions (pride), and avoid negative emotions (guilt), so that they are more likely to carry out green purchase behavior. Hence, we propose the following hypotheses:

**Hypothesis** **2** **(H2).** *Environmental commitment is positively associated with anticipated pride*.

**Hypothesis** **3** **(H3).** *Environmental commitment is positively associated with anticipated guilt*.

**Hypothesis** **4** **(H4).** *Anticipated pride is positively associated with green purchase behavior*.

**Hypothesis** **5** **(H5).** *Anticipated guilt is positively associated with green purchase behavior*.

### 2.4. The Moderating Role of Social Norms

Social norms refer to the common or the majority’s behavior in a group [57,58,59]. According to Norm Activation Theory, social norms, as external norms, have an impact on an individual’s internal norms and emotions, and individuals tend to conform to the social norms [60,61]. In the context of green purchase behavior: At a high level of the social norms, consumers perceive that most people choose to buy green products. When consumers expect to buy green products, they meet their expectations and follow social norms. Consumers realize that what they do is beneficial to the environment and in line with norms, thus resulting in a stronger sense of anticipated pride. When consumers choose not to buy green products, they feel that they fail to live up to their expectations and violate social norms. Consumers realize that what they do is harmful to the environment and goes against norms, thus resulting in a stronger sense of anticipated guilt. At a low level of social norms, consumers perceive that most people choose not to buy green products. When consumers expect to buy green products, although their actions are beneficial to the environment, they violate social norms. They feel that it makes most people to think they deliberately show off or break group norms, thus resulting in a weaker sense of anticipated pride. When consumers expect not to buy green products, although their actions are harmful to the environment, they follow social norms and are consistent with most people; they feel that it is a safe and reasonable choice not to implement green purchase behaviors, thus resulting in a weaker sense of anticipated pride.

At a high level of social norms, consumers have a certain willingness to purchase green products based on their environmental commitment, and perceive that most of them choose to buy green products, which makes consumers more aware that buying green products is the correct behavior and are more willing to implement green purchase behavior. At a low level of social norms, consumers have a certain willingness to purchase green products based on their environmental commitment, but find that most other people do not implement green purchase behavior, which makes consumers aware that non-green purchase behavior have little adverse impact on the environment, thus weakening consumers’ willingness to purchase green products and reducing the possibility of green purchase behavior. Hence, we propose the following hypotheses:

**Hypothesis** **6** **(H6).** *Social norms play a positive moderating role between environmental commitment and anticipated pride.*

**Hypothesis** **7** **(H7).** *Social norms play a positive moderating role between environmental commitment and anticipated guilt.*

**Hypothesis** **8** **(H8).** *Social norms play a positive moderating role between environmental commitment and green purchase behavior.*

**Figure 1:**
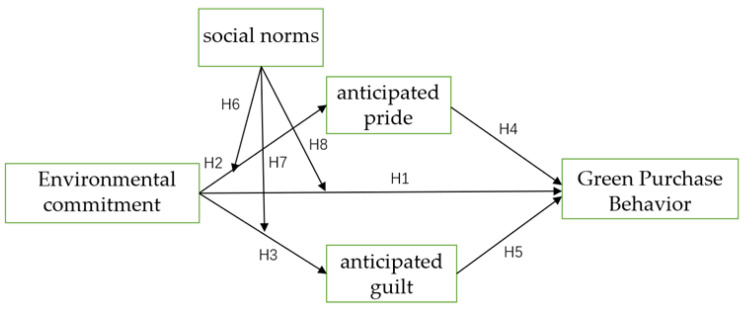
Research framework.

Based on the above, the antecedent of the research framework is environmental commitment and the consequence is green purchase behavior, while anticipated pride and anticipated guilt are mediators and social norms play a moderating role. The research framework is shown in Figure 1.

## 3. Materials and Methods

This research adopted randomized control trials and we designed one pre-study and two formal studies. The pre-study was to determine the manipulation materials used in formal studies. Study 1 was to verify the impact of environmental commitment on green purchase behavior. Study 2 was to explore the mediating role of anticipated pride and anticipated guilt and the moderating role of social norms.

This study intended to select a green product that is widely consumed and used in public life as the green product in the experimental material. Through the Internet we accessed the relevant information as well as a collection of green products appearing in the related literature on green purchase behavior, finding the following common green products: degradable shopping bags, energy-saving home appliances, new energy vehicles, recycled paper, environmentally friendly batteries, etc. Through discussions with 10 teachers and students in the school, it was agreed that degradable shopping bags are more commonly used in consumers’ daily life. At the same time, considering the limited income and consumption level of the student group, the price of degradable shopping bags is relatively low and affordable. Compared with ordinary plastic shopping bags, degradable shopping bags have the advantages of hygiene, non-toxicity, non-polluting, and can reduce carbon dioxide emissions and energy consumption. They can also automatically decompose under certain conditions, effectively reducing the white pollution brought to the environment. Promoting the purchase and use of degradable shopping bags is of great significance to preventing white pollution, reducing carbon emissions, developing green industries, and building a better living environment. Therefore, in this study we selected degradable shopping bags as the representative green product for the related experimental method.

### 3.1. Experimental Materials

#### 3.1.1. Elicitor of Environmental Commitment

We followed the experimental process of Davis et al. [7]. The subjects answered five open questions at the beginning of the experiment, and then filled in the environmental commitment measurement items to measure their current environmental commitment. Based on the Chinese cultural background, we made some modifications to the questions in the research of Davis et al. [7], thereby enhancing the effectiveness of the questionnaire. The questions in the high environmental commitment priming material were as follows: (1) If you live in a city and can’t visit the natural environment (beaches, mountains, deserts, etc.), what will you miss the most? (2) Suppose you are choosing from two possible vacations: city vacation (like Beijing) and outdoor vacation (like Zhangjiajie), what are the benefits of choosing the outdoor vacation? (3) Describe one or two ways in which you feel connected to the natural environment. What’s more, what positive effects does the environment have on you (e.g., what is good for you to be outdoors or in nature)? (4) Describe some outdoor (rather than indoor) activities you enjoy. (5) Why is it important to protect the environment? Many people try to save energy and water, why is it important for people to do so? The questions in the low environmental commitment priming material were as follows: (1) Describe two reasons why people don’t like being in a natural environment. What is the inconvenience of spending time outdoors? (2) Suppose you are choosing from two possible vacations: city vacation (like Beijing) and outdoor vacation (like Zhangjiajie), what are the benefits of choosing the city vacation? (3) Most of the things we do don’t actually improve or harm the environment. What are the things you do every day that have no impact on the environment? (4) Describe some indoor (rather than outdoor) activities you enjoy. (5) In what ways it is not convenient for you to protect the environment? For example, many people do not save energy and water, what are the reasons why people do not do so?

#### 3.1.2. Elicitor of Social Norms

The stimulus material for social norms is a piece of normative cue information. We made appropriate modifications on the basis of the research of Smith et al. [62] to make the stimulus material more compatible with the research content of this paper. In order to manipulate the social norms perceived by the subjects, the results of a school-wide survey on green product purchase behavior were provided to the subjects. Specifically, under the condition of high social norms, the prompt information obtained by the subjects was as follows: “According to the results of our survey of students in this school, about 85% of the students choose to buy degradable shopping bags after shopping in the supermarket.” Under the condition of low social norms, the prompt information the subjects got was as follows: “According to the results of our survey of students in this school, about 25% of the students choose to buy degradable shopping bags after shopping in the supermarket.”

### 3.2. Measurement of Variables

The measurement of the relevant variables was based on mature scales, among which environmental commitment was based on Davis et al. [7], social norms were based on Smith et al. [62], and anticipated pride and anticipated guilt were based on Onwezen et al. [60]. The Theory of Planned Behavior believes that an individual’s behavioral intentions can predict his behavior well [63]. Therefore, we predicted the green purchase behavior by measuring the green purchase intention of the subjects. The measurement of green purchase behavior was based on Ajzen [63]. A Likert scale was adopted, and the options were scored as “1 = completely disagree” to “7 = completely agree”. The measurement of the relevant variables is reported in Appendix A.

## 4. Results

### 4.1. Pre-Study

In the pre-study, we tested the manipulation effect of the questions in the high/low environmental commitment priming materials on the subjects’ environmental commitment level, the manipulation effect of the prompting information in social norms stimulus material on the subjects’ perceived social norm level, and the reliability of the environmental commitment scale and social norm scale.

#### 4.1.1. Participants and Procedure

In this pre-study, the recruitment information of the subjects was released on Sojump (recruitment deadline: 10 March 2022), informing that this is a survey on green product purchase behavior, and the survey is completely anonymous. Information will not be leaked, and the results are used for academic research only. A total of 60 subjects were randomly recruited, and the experiment was done over the internet.

The subjects were divided into two groups with 30 people in each group, which were the high-commitment and high-norms group and low-commitment and low-norms group. First, we informed the subjects that this is a survey on purchase behavior of green products and distributed the corresponding experimental materials to each group of subjects, including environmental commitment stimuli, environmental commitment measurement items, social norms stimuli, and social norms measurement items. The subjects need to (1) carefully read the environmental commitment stimulus material, answer five open-ended questions, and then fill in the environmental commitment measurement items according to their own feelings; (2) read the social norms prompt information, and then fill in the social norm measurement items according to their own feelings; and (3) fill in the demographic information. During the experiment, the order was strictly controlled, and the questionnaires were collected after filling in.

In total, 60 questionnaires were returned; 5 invalid questionnaires were excluded (no obvious change in options and incomplete filling), meaning 55 valid questionnaires were obtained (24 males; 31 females; mean age = 21.41 years, SD = 0.9), and the overall effective rate was 91.7%. To be specific, 28 valid questionnaires were collected from the high-commitment and high-norms group, and 27 valid questionnaires were collected from the low-commitment and low-norms group.

#### 4.1.2. Results and Discussion

The reliability of the scales was tested. Both the environmental commitment scale (eleven items, α = 0.902) and social norm scale (three items, α = 0.918) exceeded the standard value of Cronbach’s alpha (0.7), which indicates strong internal consistency of the adopted scales.

We performed a *t*-test to determine whether the priming manipulation affected participants’ scores on the environmental commitment scale. Participants in the high-commitment and high-norm group (M_high-commitment_ = 5.556, SD_high-commitment_ = 0.612) had marginally higher scores on environmental commitment scale compared to participants in the low-commitment and low-norm group (M_low-ommitment_ = 4.926, SD_low-ommiment_ = 0.384; *t* = 5.825, *p* = 0.000 < 0.001), indicating that the mean values of environmental commitment of the two groups were significantly different, showing that environmental commitment was successfully manipulated in the pre-study, and the stimulus material of environmental commitment could be used in subsequent experiments.

We performed a *t*-test to determine whether the priming manipulation affected the participants’ scores on the social norm scale. Participants in the high-commitment and high-norm group (M_high-norm_ = 5.215, SD_high-norm_ = 1.072) had marginally higher scores on the social norm scale compared to participants in the low-commitment and low-norm group (M_low-norm_ = 4.232, SD_low-norm_ = 0.515; *t* = 4.825, *p* = 0.000 < 0.001), indicating that the mean values of social norm of the two groups were significantly different, showing that social norm was successfully manipulated in the pre-study, and the stimulus material for social norms could be used in subsequent experiments.

### 4.2. Study 1

In Study 1, we tested the main effect (H1) of environmental commitment on green purchase behavior and the reliability of the related scales.

#### 4.2.1. Participants and Procedure

In Study 1, the recruitment information for subjects was released on WeChat (recruitment deadline: 20 March 2022), informing that this is a survey on green product purchase behavior, and that the survey would be completely anonymous. Information will not be leaked, and results are used for academic research only. A total of 200 subjects were randomly recruited, and the experiment was done over the internet.

The subjects were divided into two groups with 100 people in each group, which were the high-commitment group and low-commitment group. First, we informed the subjects that this is a survey on purchase behavior of green products and distributed the corresponding experimental materials to each group of subjects, including environmental commitment stimuli, environmental commitment measurement items, and green purchase behavior measurement items. The subjects needed to (1) carefully read the environmental commitment stimulus material, answer five open-ended questions, and then fill in the environmental commitment measurement items according to their own feelings; (2) fill in the green purchase behavior measurement items according to their own feelings; and (3) fill in the demographic information. During the experiment, the order was strictly controlled, and the questionnaires were collected after filling in.

In total, 200 questionnaires were returned; 20 invalid questionnaires were excluded (no obvious change in options and incomplete filling), meaning 180 valid questionnaires were obtained (85 males; 95 females; mean age = 24.39 years, SD = 0.9), and the overall effective rate was 90.0%. To be specific, 91 valid questionnaires were collected from the high-commitment group and 89 valid questionnaires were collected from the low-commitment group.

#### 4.2.2. Results and Discussion

The reliability of the scales was tested. Both the environmental commitment scale (eleven items, α = 0.913) and green purchase behavior scale (three items, α = 0.926) exceeded the standard value of Cronbach’s alpha (0.7), which indicates the strong internal consistency of the adopted scales.

We performed a *t*-test to determine whether environmental commitment affected participants’ scores on the green purchase behavior scale. Participants in the high-commitment group (M_high-commitment_ = 5.534, SD_high-commitment_ = 0.837) had marginally higher scores on green purchase behavior compared to participants in the low-commitment group (M_low-ommitment_ = 4.909, SD_low-ommiment_ = 0.789; *t* = 3.604, *p* = 0.000 < 0.001), indicating that the mean values of green purchase behavior of the two groups were significantly different, showing that there are significant differences in the impact of different environmental commitment levels on green purchase behavior. Consumers with higher environmental commitment are more willing to implement green purchase behavior. Thus, H1 is proved.

### 4.3. Study 2

In Study 2, we tested the mediating role of anticipated pride and anticipated guilt, the moderating role of social norms (H2–H8), and the reliability of the related scales.

#### 4.3.1. Participants and Procedure

In Study 2, the recruitment information for subjects was released on Weibo (recruitment deadline: 30 March 2022), informing that this is a survey on green product purchase behavior, and that the survey would be completely anonymous. Information will not be leaked, and results are used for academic research only. A total of 400 subjects were randomly recruited, and the experiment was done over the Internet.

The experiment used a 2 (environmental commitment: high, low) × 2 (social norms: high, low) between-subjects design to divide the experimental participants into four groups, with 100 people in each group, which were the high-commitment and high-norms group, low-commitment and high-norms group, high-commitment and low-norms group, and low-commitment and low norms-group. First, we informed the subjects that this is a survey on the purchase behavior of green products and distributed the corresponding experimental materials to each group of subjects, including the environmental commitment stimuli, environmental commitment measurement items, social norms stimuli, social norms measurement items, anticipated pride and anticipated guilt measurement items, and green purchase behavior measurement items. The subjects need to (1) carefully read the experimental instructions, answer five open-ended questions, and then fill in the environmental commitment measurement items according to their own feelings; (2) read the social norm prompt information, and then fill in the social norm measurement items according to your own feelings; (3) fill in the anticipated pride and anticipated guilt measurement items, as well as the green purchase behavior measurement items, according to their current feelings; and (4) fill in their demographic information. During the experiment, the order was strictly controlled, and the questionnaires were collected after filling in.

In total, 400 questionnaires were returned; 38 invalid questionnaires were excluded (no obvious change in options and incomplete filling), meaning 362 valid questionnaires were obtained (175 males; 187 females; mean age = 25.59 years, SD = 1.19), and the overall effective rate was 90.5%. To be specific, 91 in the high-commitment and high-norms group, 87 in the low-commitment and high-norms group, 90 in the high-commitment and low-norms group, and 94 in the low commitment and low norms group.

#### 4.3.2. Results and Discussion

The reliability of the scales was tested. All the scales, namely, the environmental commitment scale (eleven items, α = 0.916), social norm scale (three items, α = 0.942), anticipated pride scale (five items, α = 0.928), anticipated guilt scale (five items, α = 0.922), and green purchase behavior scale (three items, α = 0.935), exceeded the standard value of Cronbach’s alpha (0.7), which indicates the strong internal consistency of the adopted scales.

The bootstrap mediation analysis was adopted to demonstrate the mediating role of the relevant variables. The results showed (see Table 1) that (1) Coe. (X→M1) = 0.567, *t* = 4.696, Sig = 0.000 < 0.001, 95%CI (0.327, 0.806). Therefore, consumers with higher environmental commitment have higher anticipated pride, and H2 is proved; (2) Coe. (X→M2) = 0.515, *t* = 5.031, Sig = 0.000 < 0.001, 95%CI (0.316, 0.719). Therefore, consumers with higher environmental commitment have higher anticipated guilt, and H3 is proved; (3) Coe. (M1→Y) = 0.384, *t* = 4.754, Sig = 0.000 < 0.001, 95%CI (0.224, 0.546). Therefore, consumers with higher anticipated pride are more willing to implement green purchase behavior, and H4 is proved; and (4) Coe. (M2→Y) = 0.346, *t* = 3.630, Sig = 0.000 < 0.001, 95%CI (0.157, 0.536), Therefore, consumers with higher anticipated guilt are more willing to implement green purchase behavior, and H5 is proved.

The bootstrap moderation analysis was adopted to demonstrate the moderating role of the relevant variables. The results showed (see Table 2, Table 3 and Table 4) that (1) when the outcome variable is anticipated pride, Coe. (X ∗ W) = 0.467, *t* = 2.083, *p* = 0.029 < 0.05, 95%CI (0.025, 0.910). Therefore, social norms significantly moderate the relationship between environmental commitment and anticipated pride, and H6 is proved; (2) when the outcome variable is anticipated guilt, Coe. (X ∗ W) = 0.575, *t* = 3.374, *p* = 0.000 < 0.01, 95%CI (0.239, 0.915). Therefore, social norms significantly moderate the relationship between environmental commitment and anticipated guilt, and H7 is proved; (3) when the outcome variable is green purchase behavior, Coe. (X ∗ W) = 0.177, *t* = 0.692, *p* = 0.390 > 0.05, 95%CI (−0.328, 0.681). Therefore, the moderating effect of social norms on the relationship between environmental commitment and green purchase behavior is not significant, and H8 is rejected.

### 4.4. Summary of the Hypothesis Test Results

Based on the above data analysis, the study summarizes the research results. Table 5 shows the results of the research hypotheses in this study.

## 5. Discussion

Based on relevant theories, this study constructs a research model on the impact of environmental commitment on consumers’ green purchase behavior in the context of China and proposes eight research hypotheses. The results of the randomized control trials and empirical analysis show that environmental commitment significantly affects green purchase behavior; that is, consumers with stronger environmental commitment are more likely to buy green products. Furthermore, anticipated pride and anticipated guilt mediate the effect of environmental commitment on green purchase behavior. Meanwhile, social norms moderate the positive effect of environmental commitment on anticipated pride and anticipated guilt. Compared with low social norms, environmental commitment has a greater effect on anticipated pride and anticipated guilt in the case of high social norms.

### 5.1. Research Implications

The findings extend research on green purchase behavior in three important ways. First, there is no consensus on whether environmental commitment can promote green purchase behavior. This study focuses on the impact of environmental commitment on green purchase behavior in the context of Chinese culture, which will help to enrich the research on consumers’ green purchase behavior and confirms the effect of sample selection on the results to a certain extent. At the same time, it can broaden the application scope of environmental commitment in different cultural contexts. Second, scholars have rarely explored the internal mechanism of environmental commitment affecting consumers’ green purchase behavior. This study explored the mediating effect of anticipated pride and anticipated guilt on the impact of environmental commitment on consumers’ green purchase behavior and the moderating role of social norms through randomized control trials. It helps to open the black box of the relationship between environmental commitment and consumers’ green purchase behavior, to understand how environmental commitment affects green purchase behavior. Third, this study contributes to past research by exploring the moderating role of social norms in green purchase behavior. Specifically, this study provides empirical evidence that, under a condition of high social norms, the positive effects of environmental commitment on anticipated pride and anticipated guilt are enhanced, ultimately promoting green purchase behavior.

### 5.2. Managerial Implications

This study has practical implications for policy and marketing strategy makers. Governments and enterprises should take into account the important role of environmental commitment when propagandizing green consumption pattern and promoting consumers’ green purchase behavior. They could emphasize to consumers the inseparable and interdependent relationship between mankind and natural environment, subtly improving the level of consumers’ environmental commitment, thereby promoting green purchase behavior. They also should pay attention to the influence of consumers’ inner emotions on their green purchase behavior, and convey to consumers the vital impact of green purchase behavior on natural environment, thus improving consumers’ sense of pride in implementing green purchase behavior and sense of guilt in non-green purchase behavior. Moreover, governments and enterprises should consider the significant impact of social norms on consumers’ internal psychology, showing more practical cases of green purchase behavior to consumers in policy publicity and marketing promotion, and control the dissemination of information on non-green purchase behavior, strengthening consumers’ perception of the social norm that most people choose green purchase behavior, thereby promoting consumers’ green purchase behavior.

### 5.3. Limitations and Future Research

Although three experiments were performed in this study to ensure the reliability of the results, there are still some limitations that need to be addressed. First, the only green product selected in the experimental materials is degradable shopping bags, and this category of green products is relatively simple. In future experiments, different categories of green products can be selected as experimental products. Second, the measurement of the subjects’ green purchase behavior in the experiment is based on the behavioral willingness of the subjects, and there may be a certain gap with the actual purchase behavior. In future research, field experiments can be carried out to monitor the actual purchase behavior of consumers more intuitively, and the obtained data may be more realistic and reliable than that in the laboratory environment. Third, the use of questionnaires can generate a problem that may affect the relevance of the research, known as the bias effect or common method bias (CMB). Such problems arise when data on independent and dependent variables emanate from the same respondent and the same measurement scale exists throughout the questionnaire. Future research should avoid the influence of common method bias. Additionally, scholars can explore more mediating variables, such as perceived value, and different moderating variables, such as product types, so as to expand the depth and breadth of research on the impact of environmental commitment on consumers’ green purchase behavior in the context of Chinese culture.

## 6. Conclusions

There is no consensus on whether environmental commitment can promote green purchase behavior. Especially in the Chinese context, the validity and internal mechanism of the impact of environmental commitment on green purchase behavior have not been deeply studied. This study explores the influence mechanism of environmental commitment on consumers’ green purchase behavior. Results reveal that environmental commitment significantly promotes green purchase behavior. Anticipated pride and anticipated guilt mediate the relationship between environmental commitment and green purchase behavior. Social norms moderate the positive effect of environmental commitment on anticipated pride and anticipated guilt. Compared with low social norms, environmental commitment has a greater effect on anticipated pride and anticipated guilt in the case of high social norms.

This study focuses on the impact of environmental commitment on green purchase behavior in the context of Chinese culture, which helps to enrich the research on consumers’ green purchase behavior and broaden the application scope of environmental commitment in different cultural contexts. Moreover, it contributes to past research by exploring the moderating role of social norms in green purchase behavior. The findings could guide governments and marketers to value consumers’ environmental commitment, focus on the role of emotions, and pay attention to the impact of social norms on green consumption.

## Figures and Tables

**Table 1:** Path coefficients.

Path	Coe.	S.E.	T	Sig	LLCI	ULCI
X→M1	0.567	0.119	4.696	0.000	0.327	0.806
X→M2	0.515	0.103	5.031	0.000	0.316	0.719
M1→Y	0.384	0.082	4.754	0.000	0.224	0.546
M2→Y	0.346	0.0935	3.630	0.000	0.157	0.536

Notes: M1 = anticipated pride; M2 = anticipated guilt; X = environmental commitment; Y = green purchase behavior.

**Table 2:** The moderating role of social norms (1).

Moderator	M1						
		Coe.	S.E.	*t*	*p*	LLCI	ULCI
Social Norms	X	0.349	0.156	2.236	0.025	0.041	0.658
	W	0.340	0.159	2.147	0.030	0.027	0.655
	X ∗ W	0.467	0.224	2.083	0.029	0.025	0.910

Notes: M1 = anticipated pride; X = environmental commitment; W = social norms.

**Table 3:** The moderating role of social norms (2).

Moderator	M2						
		Coe.	S.E.	*t*	*p*	LLCI	ULCI
Social Norms	X	0.249	0.121	2.093	0.037	0.014	0.483
	W	0.426	0.123	3.534	0.000	0.188	0.667
	X ∗ W	0.575	0.171	3.374	0.000	0.239	0.915

Notes: M2 = anticipated guilt; X = environmental commitment; W = social norms.

**Table 4:** The moderating role of social norms (3).

Moderator	Y						
		Coe.	S.E.	*t*	*p*	LLCI	ULCI
Social Norms	X	0.149	0.175	0.836	0.405	−0.200	0.495
	M1	0.368	0.085	4.365	0.000	0.203	0.537
	M2	0.300	0.111	2.708	0.008	0.083	0.518
	W	0.016	0.183	0.085	0.835	−0.344	0.373
	X ∗ W	0.177	0.255	0.692	0.390	−0.328	0.681

Notes: M1 = anticipated pride; M2 = anticipated guilt; X = environmental commitment; W = social norms; Y = green purchase behavior.

**Table 5:** Summary of the results for each hypothesis.

Hypothesis	Description	Result
H1	Environmental commitment is positively associated with green purchase behavior.	Supported
H2	Environmental commitment is positively associated with anticipated pride.	Supported
H3	Environmental commitment is positively associated with anticipated guilt.	Supported
H4	Anticipated pride is positively associated with green purchase behavior	Supported
H5	Anticipated guilt is positively associated with green purchase behavior.	Supported
H6	Social norms play a positive moderating role between environmental commitment and anticipated pride.	Supported
H7	Social norms play a positive moderating role between environmental commitment and anticipated guilt.	Supported
H8	Social norms play a positive moderating role between environmental commitment and green purchase behavior.	Not supported

## Data Availability

The data that support the findings of this study are available from the corresponding author, upon reasonable request.

## Appendix A

**Table A1:** The information of the scales.

Constructs	Numbers	Content	Resources
Environmental commitment	EC1	I am interested in strengthening my connection with the environment in the future.	Davis et al. [7]
	EC2	I feel strongly linked to the environment.	
	EC3	When I make plans for myself, I take into account how my decisions may affect the environment.	
	EC4	It seems to me that humans and the environment are interdependent.	
	EC5	It makes me feel good when something happens that benefits the environment.	
	EC6	Feeling a connection with the environment is important to me.	
	EC7	I expect that I will always feel a strong connection with the environment.	
	EC8	I believe that the well-being of the natural environment can affect my own well-being.	
	EC9	It is unlikely that I’ll feel a connection to the environment in the future. (R)	
	EC10	I feel very attached to the natural environment.	
	EC11	I feel committed to keeping the best interests of the environment in mind.	
Social norms	SN1	Most of the people who are important to me bought degradable shopping bags after shopping at the supermarket.	Smith et al. [62]
	SN2	Most of the people in our school bought degradable shopping bags after shopping at the supermarket.	
	SN3	Most of my friends bought degradable shopping bags after shopping at the supermarket.	
Anticipated pride	AP1	If I buy degradable shopping bags in the future, I will feel proud.	Onwezen et al. [60]
	AP2	If I buy degradable shopping bags in the future, I will feel a sense of achievement.	
	AP3	If I buy degradable shopping bags in the future, I will feel confident.	
	AP4	If I buy degradable shopping bags in the future, I will feel satisfied.	
	AP5	If I buy degradable shopping bags in the future, I will feel it’s worthwhile.	
Anticipated guilt	AG1	If I don’t buy degradable shopping bags in the future, I will feel guilty.	Onwezen et al. [60]
	AG2	If I don’t buy degradable shopping bags in the future, I will feel regret.	
	AG3	If I don’t buy degradable shopping bags in the future, I will feel sorry.	
	AG4	If I don’t buy degradable shopping bags in the future, I will feel bad.	
	AG5	If I don’t buy degradable shopping bags in the future, I will feel ashamed.	
Green purchase behavior	GPB1	If I need to buy shopping bags at the supermarket in the future, I will buy degradable bags.	Ajzen [63]
	GPB2	If I need to buy shopping bags at the supermarket in the future, I intend to buy degradable bags.	
	GPB3	Imagine you’ve just finished shopping at the supermarket, and you need to buy a shopping bag at checkout, you will choose to buy a degradable bag	
